# Curves for Curlew: Identifying Curlew breeding status from GPS tracking data

**DOI:** 10.1002/ece3.9509

**Published:** 2022-12-12

**Authors:** Katharine M. Bowgen, Stephen G. Dodd, Patrick Lindley, Niall H. K. Burton, Rachel C. Taylor

**Affiliations:** ^1^ British Trust for Ornithology Cymru Bangor UK; ^2^ British Trust for Ornithology, The Nunnery Thetford, Norfolk UK; ^3^ RSPB Centre for Conservation Science, The Lodge Sandy, Bedfordshire UK; ^4^ Natural Resources Wales Bangor UK

**Keywords:** Curlew, GPS tracking, nest monitoring, *Numenius arquata*, recurse package, shorebirds, waders

## Abstract

Identifying the breeding status of cryptic bird species has proved problematic without intense or inherently expensive monitoring. Most, if not all, intensive bird monitoring comes with disturbance risks and many projects now rely on tagging of individuals to provide remote information on movements. Given the importance of breeding status when targeting conservation interventions novel methods are needed. This study aimed to identify breeding status in Eurasian Curlew (*Numenius arquata*) from GPS tag movement patterns using the “*recurse”* package in R. This package identifies foci of activity (using *K*‐means clustering) based on revisitations. Using a training data set from an individual of known breeding status, we visually assessed the frequency of revisits to particular locations to identify prebreeding, incubation, chick guarding, and post‐breeding stages to an accuracy of a within at most half a day and thus breeding outcomes. Limited validation was provided by additional field observations. Based on our results, we estimate a low daily nest survival rate of 0.935 during incubation, that only a small proportion of individuals successfully raised young, and that there was a high proportion (26%) of non‐breeders in the population. The Eurasian Curlew is a species of high conservation concern across Europe, and our results concur with recent studies highlighting that population declines are likely to be driven by low levels of productivity. The acquisition of improved knowledge on the behaviors of individuals at each stage of breeding enables more targeted conservation efforts and reduces the need for additional monitoring visits that may cause increased disturbance and risk of nest failure. We hope that the approach outlined may be developed to provide practitioners who have detailed knowledge of the behavior of their study species with a practical means of assessing the breeding status and outcomes of their study populations.

## Graphical abstract

Our study has found that it is possible to identify breeding stage of Curlew in terms of incubation, chick rearing or failure from the movement patterns of GPS tagged birds alone. By using frequency plots from a package that normally just determines clustering of points, we can remotely identify the successes and failures of cryptic species that normally wouldn't be possible without intensive and expensive fieldwork.

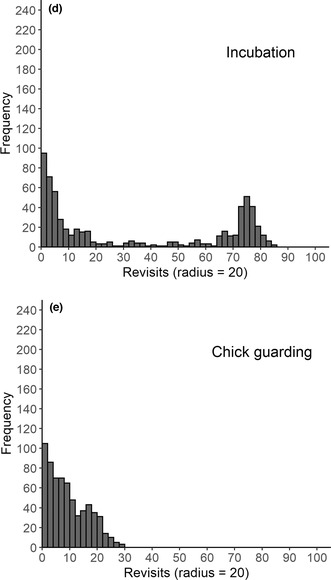

## 1 INTRODUCTION

Remote tracking and tagging of animals is becoming ever more widespread across zoological research programs (Casper, 2009), aided by the increasing miniaturization of many devices that has enabled their application to a greater range of target species (Portugal & White, 2018). While the main purpose of the majority of tagging programs is to collect locational data to understand movement and area use, both locational data and the information collected through additional sensors, e.g., accelerometers or heart‐rate and temperature loggers, may be used to infer behavior, maximizing the output from placing interventions onto individuals (Ropert‐Coudert & Wilson, 2005; Wilmers et al., 2015).

Understanding breeding behaviors and outcomes is vital when putting together conservation plans and management. While the causes of species declines can occur throughout a species' life history, maintaining rates of recruitment remains important to ensure stable or increasing populations. Nevertheless, fledging estimates are missing or unknown for many species especially breeding waders (Charadriiformes, “shorebirds” in North America) where the precocial nature of their offspring makes monitoring of breeding attempts difficult after incubation (Lukacs et al., 2004) and the point of hatching is often only reliably monitored through nest cameras or thermal loggers. For many species, monitoring the status of individual breeding attempts and consequently breeding outcomes is problematic without intensive fieldwork (Brown & Shepherd, 1993) which is often expensive, both in terms of money and person resources. Terrain and cryptic behaviors can make this almost impossible for some species (Brown & Shepherd, 1993; Valle & Scarton, 2019). In addition, such monitoring carries a risk of disturbance and therefore potential impacts on breeding outcomes (Champagnon et al., 2019; Götmark, 1992; Syrová et al., 2020). Fortunately, the rapid development of remote tracking studies provides an alternative means of monitoring species' behaviors at all stages of their life cycles (McKinnon & Love, 2018). The data from such studies can complement and add to those obtained from traditional fieldwork, with a hands‐off approach that limits impacts on study species.

The Eurasian Curlew (*Numenius arquata*; hereafter “Curlew”) is an iconic breeding species of lowland and upland grasslands and heaths, but which is rapidly declining across many regions in Europe (Young et al., 2020). It is listed as Near Threatened by BirdLife International (2020) and is Red‐listed on the United Kingdom (Gilbert et al., 2021), Wales (Johnstone & Bladwell, 2016), and Irish (Gilbert et al., 2021) Birds of Conservation Concern due to the species breeding declines. The Curlew has consequently been highlighted as an urgent conservation priority in the UK, Wales, and Ireland (Brown et al., 2015; Gylfinir Cymru/Curlew Wales, 2021; O'Donoghue et al., 2019). In the UK, a recent audit of thirty one Curlew conservation projects reinforced the need for contemporary guidance for survey methods in particular to determine breeding success (Wilson et al., 2020).

Recent studies have highlighted that the species population decline is likely to be driven by low levels of breeding productivity and subsequent recruitment, with habitat loss, predation, and human disturbance as factors within this (Brown et al., 2015; Cook et al., 2021; Franks et al., 2017; Taylor et al., 2020). Adults can survive up to 30 years (Robinson, 2005) and though studies have shown that there was an improvement in survival following the implementation of a hunting ban in the UK in 1982, this has provided only marginal improvement on the population's trajectory, with the latest estimate placing adult survival in the UK at 89.9% (Cook et al., 2021; Taylor & Dodd, 2013). For both Curlew and other declining wader species, management interventions (e.g. fencing, predator control, head starting) aimed at improving success at each breeding stage (from incubation, through the initial days post‐hatching to fledging) have been shown to have the potential for increasing recruitment into populations (Clark et al., 2018; Franks et al., 2018; Oosterveld et al., 2011). As a result, focusing research on the factors affecting success at each stage of breeding is crucial to understand the species' needs that in turn can be led into appropriate management and protection.

Global Positioning Satellite (GPS) tags and other tagging sensors (e.g., geolocators, radio transmitters, passive integrated transponder, accelerometers) have previously been used to identify the general movements, habitat use, and behavior of a large number of bird species (Arbeiter et al., 2017; Bulla et al., 2016; Ewing et al., 2018; Kosztolányi & Székely, 2002; Shamoun‐Baranes et al., 2012; Verhoeven et al., 2020). For mammals, tracking data have also been used to assess similar aspects of species ecology as well as parturition and neonate survival (Bonar et al., 2018; DeMars et al., 2013; Nicholson et al., 2019). Tracking data have been used to identify the nesting attempts of birds and their outcomes by Picardi et al. (2020) (who implemented their method in the R package “*nestR”*, Picardi et al., 2022) for wood stork (*Mycteria americana)*, lesser kestrel (*Falco naumanni*), Mediterranean gull (*Icthyaetus melanocephalus*), and by Schreven et al. (2021) for pink‐footed geese (*Anser brachyrhynchus*); these studies set the precedent for our study. With respect to waders, tracking studies have specifically been used to distinguish and assess the length of nesting (incubation) attempts in black‐tailed godwit (*Limosa limosa*; Verhoeven et al., 2020), but to the authors' knowledge, GPS data have not specifically been used to identify breeding status once young have left the nest. While the miniaturization of tagging devices provides more opportunities for deployment (Portugal & White, 2018), the trade‐off between data resolution, performance, and battery life (Mitchell et al., 2019) may be a factor in the lack of studies to date that have attempted to differentiate breeding status using tracking data.

In this study, we aimed to assess if breeding status and consequently breeding outcomes (success or failure of nesting (incubation) and overall breeding) can be determined from locational data using a preexisting R package, *“recurse”* (Bracis et al., 2018). In addition, we investigated the data resolution required for the approach outlined to work effectively for Curlew to aid in efficient planning and programming of tag schedules for other projects.

## 2 MATERIALS AND METHODS

### 2.1 Data collection and cleaning

Data were collected during a field project based at three locations in North Wales, UK (Anglesey, Snowdonia, and the Clwydian Range) during the spring breeding seasons of 2016, 2018 and 2019. North Wales contains the majority of the Welsh breeding population of Curlew, with breeding pairs found across a wide variety of habitats from lowland farmed landscapes, e.g., on the Isle of Anglesey, through to upland grassland and heath on the mainland, e.g., in Snowdonia and the Clwydian Ranges (Balmer et al., 2013; Gylfinir Cymru/Curlew Wales, 2021).

A total of 26 adult Curlew (seven females, 19 males from 25 pairs) were caught by cannon‐netting at the start of the breeding season, between 9th April and 16th May, generally prior to the start of incubation. Each bird caught was fitted with a numbered metal ring and unique combination of color rings for subsequent field identification, and morphometric measurements were taken (bill length, weight, and wing length). Sexes were determined based on bill length and weight (Summers et al., 2013). Each bird caught was fitted with a remotely downloading PathTrack Ltd nanoFix‐GEO+RF GPS tag (www.pathtrack.co.uk). Tags were on average 4.82 g in weight (<0.82% of the birds' body weight, mean = 698.3 g, range = 600–900 g) and were fitted with a small piece of gauze to aid attachment. Following the methods of Seward et al. (2021), the transmitters were attached to a small area of clipped feathers on the birds' upper backs (notarium) using cyanoacrylate glue. In the UK, ringing is licensed by the British and Irish Ringing Scheme under the Wildlife and Countryside Act 1982, as amended by the Environmental Protection Act 1990 and the Wildlife (Northern Ireland) Order 1985, with deployment of tracking technologies approved by the scheme's independent Special Methods Technical Panel (SMTP); approvals require that potential effects of deployments are appropriately monitored and reported. All activities were carried out by individuals with full UK ringing permits and appropriate endorsements approved by the SMTP. The tags remained attached for an average of 42.3 days (range 0.3–96.1, median 47.3 days) and the majority detached as expected during post‐breeding molt but a few stopped transmitting earlier due to software/battery issues. Post‐tagging all birds was observed in the field, and no impacts (in terms of altered behavior) were seen in comparison to other nontagged control birds caught and color ringed at the same time.

Tags provided locational data every 15 min (24 h a day), which were downloaded to nearby base stations whenever the birds were in range throughout the tagging period. In total, analyzable data were obtained for 23 individuals (see Table 1). The three other tagged birds that did not provide any data were present and seen in subsequent years, but the tags did not communicate with the base stations.

**TABLE 1:** Details of tagged Curlew from 3 years (2016, 2018, and 2019) in three areas of north Wales, UK.

Year	Location	Tag ID	Sex	N days of data	Number of relocations	Start (day of tagging)	End (last day of data)	Mean distance (tagging location)	Max distance (tagging location)	Mean distance during incubation
2016	Snw	303O_NN	M	30.3	2796	07/05/2016	06/06/2016	2.22	7.17	NA
		301O_NG	M	16.1	1512	08/05/2016	24/05/2016	1.54	2.03	0.11
		285O_NO	M	26.7	2530	08/05/2016	04/06/2016	2.28	12.00	0.16
2018	Snw	341O_RR	M	60.2	2108	11/05/2018	10/07/2018	16.87	40.75	NA
		355O_RG	F	47.3	2946	13/05/2018	29/06/2018	1.48	4.45	0.24
		350O_BB	M	NA	NA	13/05/2018	Not enough data – observed in the field in 2020			
		352O_OO	M	23	1744	16/05/2018	08/06/2018	1.71	2.12	0.07
	Ang	339O_OW	M	96.1	9069	05/05/2018	09/08/2018	3.31	6.89	0.13
		340O_GG	M	0.3	31	12/05/2018	12/05/2018	3.73	11.44	NA
2019	Snw	354W_BO	F	51.4	4641	17/04/2019	08/06/2019	1.56	4.36	0.58
		346W_OW	F	25.7	2377	18/04/2019	14/05/2019	1.39	1.93	0.25
		709W_RB	M	69.4	6422	18/04/2019	26/06/2019	2.08	21.91	0.70
		016W_GO	F	16.4	1431	18/04/2019	04/05/2019	2.16	4.02	NA
		351O_RW	M	67.3	6219	18/04/2019	24/06/2019	1.77	16.41	0.22
		348W_OG	M	31	1045	23/04/2019	24/05/2019	1.75	10.04	0.41
		347W_OR	F	61	5302	23/04/2019	23/06/2019	4.80	27.78	NA
		342W_BW	M	24.5	2273	23/04/2019	18/05/2019	1.43	4.11	NA
	Ang	007O_BW	F	50.1	4244	09/04/2019	29/05/2019	1.38	2.32	0.24
		087O_RN	M	58.7	5263	09/04/2019	07/06/2019	1.34	2.25	0.18
		052O_GR	M	47.8	4401	10/04/2019	28/05/2019	1.71	2.68	0.19
		085O_RO	M	43.3	3507	11/04/2019	24/05/2019	2.31	14.61	0.92
		211O_OW	M	34.6	3215	13/04/2019	17/05/2019	2.12	3.45	0.12
		041O_BO	F	NA	NA	17/04/2019	No data – bird not observed since soon after tagging			
	Clw	002O_GO	M	59.6	5144	02/05/2019	30/06/2019	5.49	9.27	0.57
		165W_RR	M	54.8	4916	02/05/2019	26/06/2019	2.71	9.22	0.73
		678O_RB	M	NA	NA	03/05/2019	No data – observed regularly in the field in 2019			

*Note*: Birds with analyzable data shown with days of tagging data downloaded, start and end of tagging periods and distances (km) traveled from analysis of each bird. The areas are indicated by Ang, Anglesey; Clw, Clwydian Ranges; Snw, Snowdonia.

Following download, the raw data were processed in PathTrack Archival USB software (Archival USB v1.44, PathTrack, UK) and then imported into R (R Development Core Team, 2020). Duplicate fixes (created from duplicate downloads to different base stations) and fixes obtained from fewer than five satellites were removed (to minimize the potential for erroneous positions). We also determined whether each relocation was during the day or night based on sunrise and sunset times (crepuscular dawn and dusk) defined by the “*maptools”* package in R (Bivand & Lewin‐Koh, 2015).

### 2.2 Breeding stage analysis

The R package “*recurse”* (Bracis et al., 2018) is designed to analyze trajectory data to look for “revisitations” (returns to an area previously visited). It calculates foci of activity (using *K*‐means clustering) for a set of relocations (defined in this study by individual) within a predefined radius which for this analysis was set at 20 m (chosen based on the average error margin of the tags used—see Appendix 1). This radius is moved along the tracks of relocations to identify the passage in and out of this area by the tagged individuals and assesses time spent inside as well as frequency of revisits.

The package was initially used simply to identify coordinates of potential nesting locations. However, we then used the “*getRecursions”* function to plot the frequencies of visits to individual locations with the aim of defining each breeding status. Given the relatively high frequency of the tracking data, we found that this function could be applied to a subset of relocations ranging from a week of data (~672) or even 3 days of data (~288) to enable the date of transition between statuses to be identified.

#### 2.2.1 Initial breeding stage training based on known status male Curlew

*Stage 1: Weekly frequency plots: Histograms of revisitation* frequency were plotted for each week of the year for which data were obtained for a male Curlew (tag ID 339O_OW) whose breeding status was known through field observations throughout the 2018 breeding season to visually define the distributions associated with breeding status. It was predicted that during incubation bird's activities would be more concentrated with repeated visits to a number of locations, most especially in and around the nest location. Both male and female Curlew share incubation duties (Billerman et al., 2020) and so relocations when birds are breeding will have a strong focal area that is revisited at a high frequency. The distributions of nonincubating birds are more spread out with high numbers of single visit relocations. Areas of higher focal intensity can be seen in the distributions of some individuals that use specific overnight or foraging locations, although these are not as clear as those shown when birds are incubating. Movement patterns of chick guarding adults were unknown as chicks are highly mobile and would only be recorded in males which do the majority of chick guarding. Failures at both the incubation and chick guarding stages were expected to be associated with the loss of previously strong patterns with clear dispersion of movements and no discernible focal areas.

*Stage 2: Identification of nest sites and dates of change*: Having identified periods of interest, we then considered data on a rolling 3‐day window basis to identify the location of the nest site, the start and end of incubation, chick guarding and the success or failure of nesting (incubation), and overall breeding attempts. This 3‐day window was identified as the minimum set of data needed to clearly identify change in breeding status for our 15 min relocations. Incubation and chick‐rearing periods were expected to be ~28 and ~35 days, respectively (Robinson, 2005). Where a change of breeding status was predicted, a detailed plot of all relocations within the 3‐day period, specified to each hour, was visually investigated to identify a specific day and approximate hour when the change occurred. Specific coordinates for nest locations were identified by applying the “*popCluster”* function in *recurse* which uses *K*‐means clustering to the top 20% of locations y number of revisits (Bracis et al., 2018).

#### 2.2.2 Application to identify breeding stages of other tagged birds

The same process was then followed to predict the breeding status of all remaining Curlew, first using the distribution patterns from the training data set above to identify periods of interest, before then identifying specific points of change between breeding stages by focussing on data within rolling 3‐day windows. We additionally aimed to validate the predicted breeding status of individuals through observations of chick rearing where possible.

Based on the results of predictions, we undertook an analysis using the Mayfield approach (Mayfield, 1975) in the programme MARK (White & Burnham, 1999) to estimate a daily nest survival rate for the incubation period.

### 2.3 Data requirements for breeding stage identification

Our analysis was based on high‐resolution GPS data, with relocations every 15 min, 24 h a day, enabling revisitations and consequently breeding status to be determined with a minimum of 3 days' data (~288 locations). However, many studies only use hourly data or less when investigating migration and others only use short bursts of data (Seward et al., 2021). To test minimum data requirements for determining a different breeding status, the full data set of relocations for male 339O_OW was reduced to samples taken at (i) 30 min, (ii) 60 min and during, and (iii) daylight mornings (08:00–13:59 UTC). We then followed the same procedure outlined above to assess how the resolution of data affected our ability to determine breeding status.

## 3 RESULTS

### 3.1 Overall data analysis available and tracking paths of tagged birds

**FIGURE 1:**
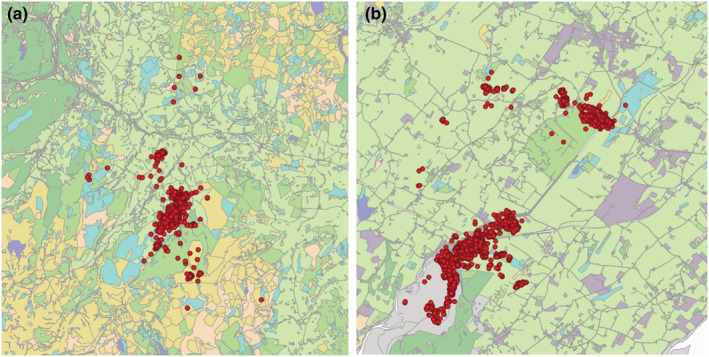
Example maps of tag relocations for two Curlew (Numenius arquata) (a) 303O_NN in 2016 in Snowdonia, and (b) 339O_OW in 2018 on Anglesey (Scale 1:80,000 and 1:60,000, respectively). The Phase 1 habitat maps (Natural Resources Wales & Countryside Council for Wales, 2019) for each area are shown beneath the relocations (dark green = forest, mid green = semi/unimproved grassland, lime/pale green = improved grassland, peach = dry scrub and heath, cyan = wet habitats (flushes, mires, peat, bog, wet grass, and heath), blue = waterbodies, orange = non accessed land or mosaic, and purple = other anthropogenic habitats).

Around ~83,000 relocations were collected for the 23 tagged birds that provided data, an average of 3615 relocations per bird (range 31–9069) and 43.3 days of data (range 0.3–96.1 days) between April and August (example relocations shown in Figure 1). The furthest distance that a bird was recorded from the tagging location was 40.75 km, while the average maximum was 9.6 km, while the maximum distance between 15 min locations for any individual was 13.3 km and 6.1 km on average across individuals (Table 1 gives individual values).

### 3.2 Initial breeding stage training based on known status male Curlew

The breeding status of male 339O_OW, tagged in 2018 was followed through that breeding season until failure at the chick stage. The regular data downloads revealed that it was focused on a particular part of a field near the tagging site, the nest cup was found and a temperature logger placed. Ad hoc observations by local conservationists and land managers during May 2018 identified the presence of hatched chicks around 4 weeks later. Within 10 days of hatching (based on analysis below) on June 13, 2018, the field that the chicks was known to use with their parent was mown and following this the male Curlew showed behavioral signs of chick loss. Attempts to find the nest cup following this were successful, but the temperature logger was not recovered.

#### 3.2.1 Stage 1: Weekly frequency plots

**FIGURE 2:**
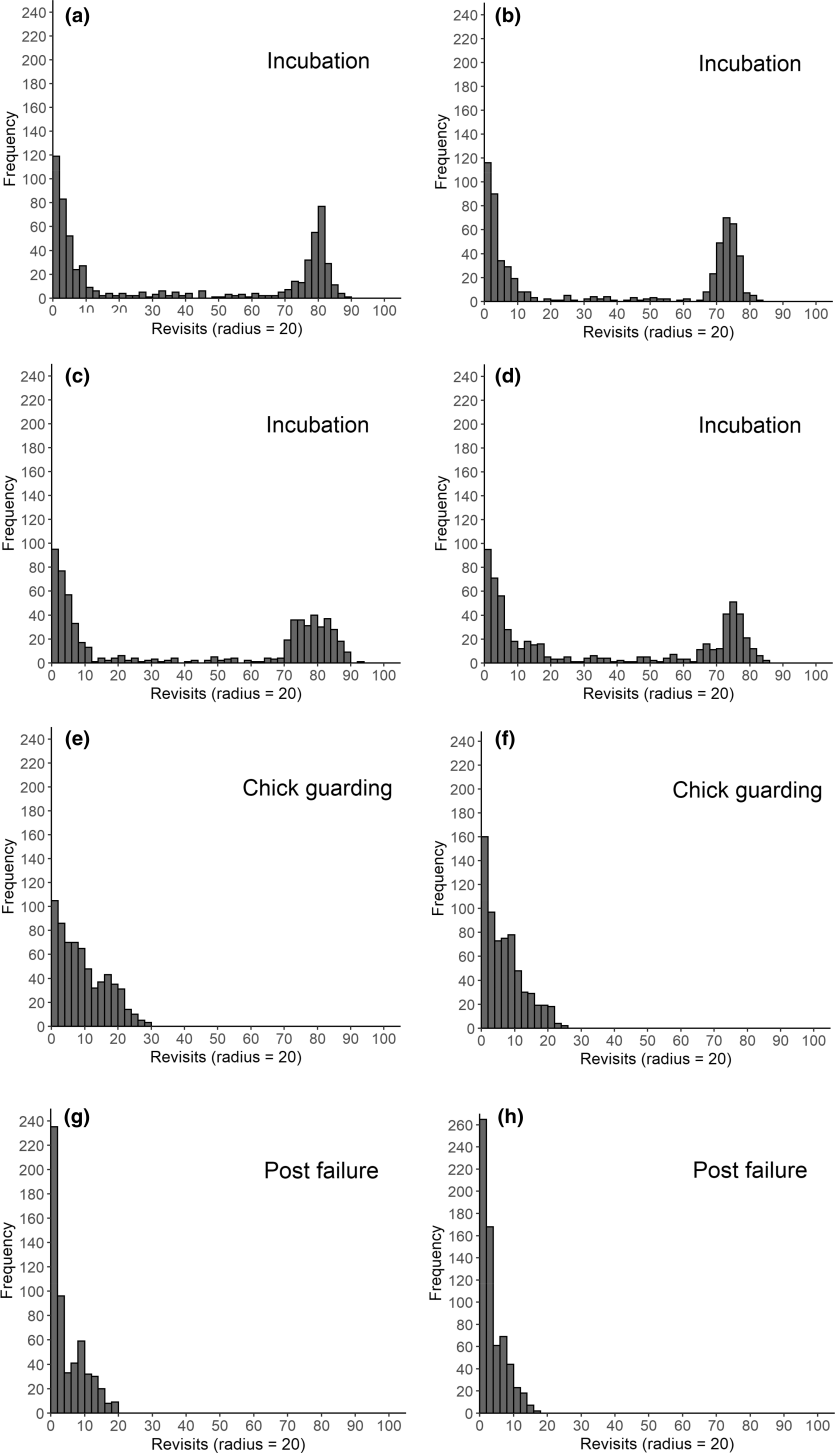
Weekly “recurse” frequency plots for male 339O_OW of known breeding status in 2018, showing the frequency of revisits to sites within a moving 20 m radius. In (a) Week 19 (May 7th–13th 2018), high numbers of revisits to particular locations suggest incubation, with this pattern continuing in (b) Week 20, (c) Week 21, and (d) Week 22. Subsequently, the more sloped distribution in the number of revisits to particular locations in (e) Week 23 suggests chick guarding, which continued into (f) Week 24 when failure occurred. Subsequently, in (g) Week 25 and (h) Week 26, a flatter and left skewed pattern was observed, which continued for the rest of the tagging period.

The frequency plots for weeks 19–22 (Figure 2a–d) are bimodal showing that the first peak represents sites visited infrequently (likely to be foraging sites), while the second peak is for sites with higher numbers of repeated visits that are likely to be at and around the nesting site, indicating incubation. In week 23 (Figure 2e), the sloped pattern indicates when the male was chick guarding—with many places visited a few times, but no single foci, reflecting that chicks were mobile but not capable of traveling far. In week 24 (Figure 2f), when chicks were lost, the male visited more sites just a single time; locational data showed the male moving further away as it gave up on the breeding field. In weeks 25–26, post‐failure, the male moved to the nearby estuary while still occasionally visiting the breeding site and the plots indicate a high number of places only ever visited once with no strong foci.

#### 3.2.2 Stage 2: Identification of nest sites and dates of change

**FIGURE 3:**
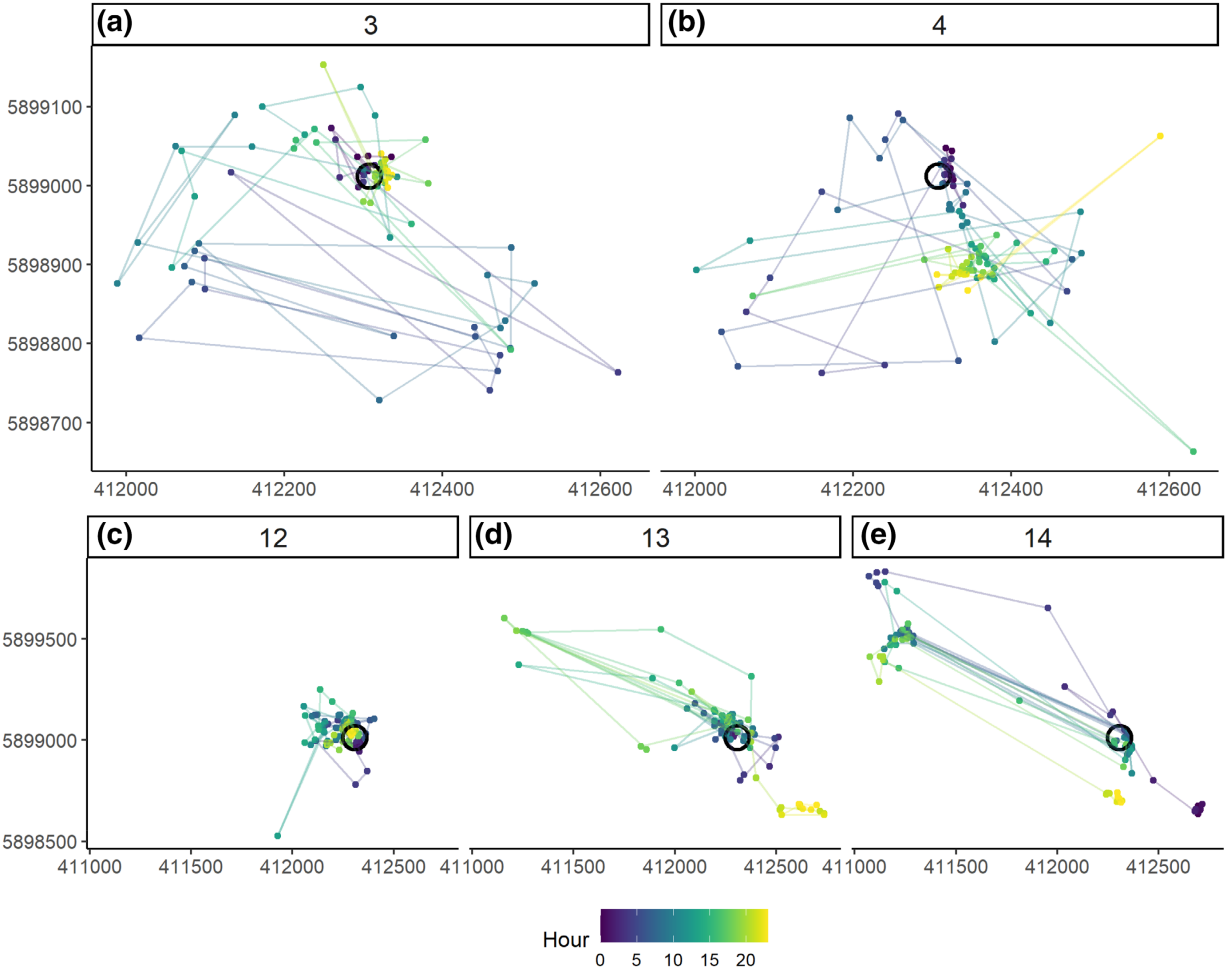
“Recurse” locational plots for a male Curlew (Numenius arquata) 339O_OW of known breeding status in 2018, indicating the change from incubation to chick guarding between the (a) 3rd and (b) 4th June where the relocations slowly move south of the nest site (sharing of incubation duties observable from male's prehatching movements) and (c–e) the days around the date of chick loss (d – June 13, 2018) showing the subsequent scattering of locations visited. In all plots colors indicate the hour of the day, starting at dark purple just after midnight 00:00 h through blue and green to yellow at 23:00. The nest location is indicated by a black circle on each plot and lines indicate movement between sequential locations.

The plots in Figure 2 were constructed using 7 days of data, which enabled general patterns associated with breeding status to be identified, but not the specific dates at which changes occurred. To identify the specific point of change between each breeding status, the *getRecursions* function was run over the smallest subset of data possible without error which proved to be 3 days (~288 relocations). By focussing on those weeks where change occurred, with a rolling 3 day window of data, we identified the specific timing of change based on plots of timed locational data (Figure 3). On 3rd June (Figure 3a) focused locations in one area in both the early and late hours of the day indicate the nest location; on 4th June (Figure 3b) the slow movement of the centre of focus away from this area indicates mobile chicks (Curlew chicks are precocial within hours of hatching and they move away from the hatching location, guarded by an adult, Currie et al., 2001). This movement occurs during the early afternoon indicating the chicks hatched in the early to mid‐morning. Chick failure was known to occur on the afternoon of 13th June (Figure 3d) after which there is a movement to areas not previously used (Figure 3e).

Nest site coordinates were specifically identified using the *popCluster* function on the subset of relocations identified as being associated with incubation from the analysis above (Figure 3).

### 3.3 Application to identify breeding status of other tagged individuals

**FIGURE 4:**
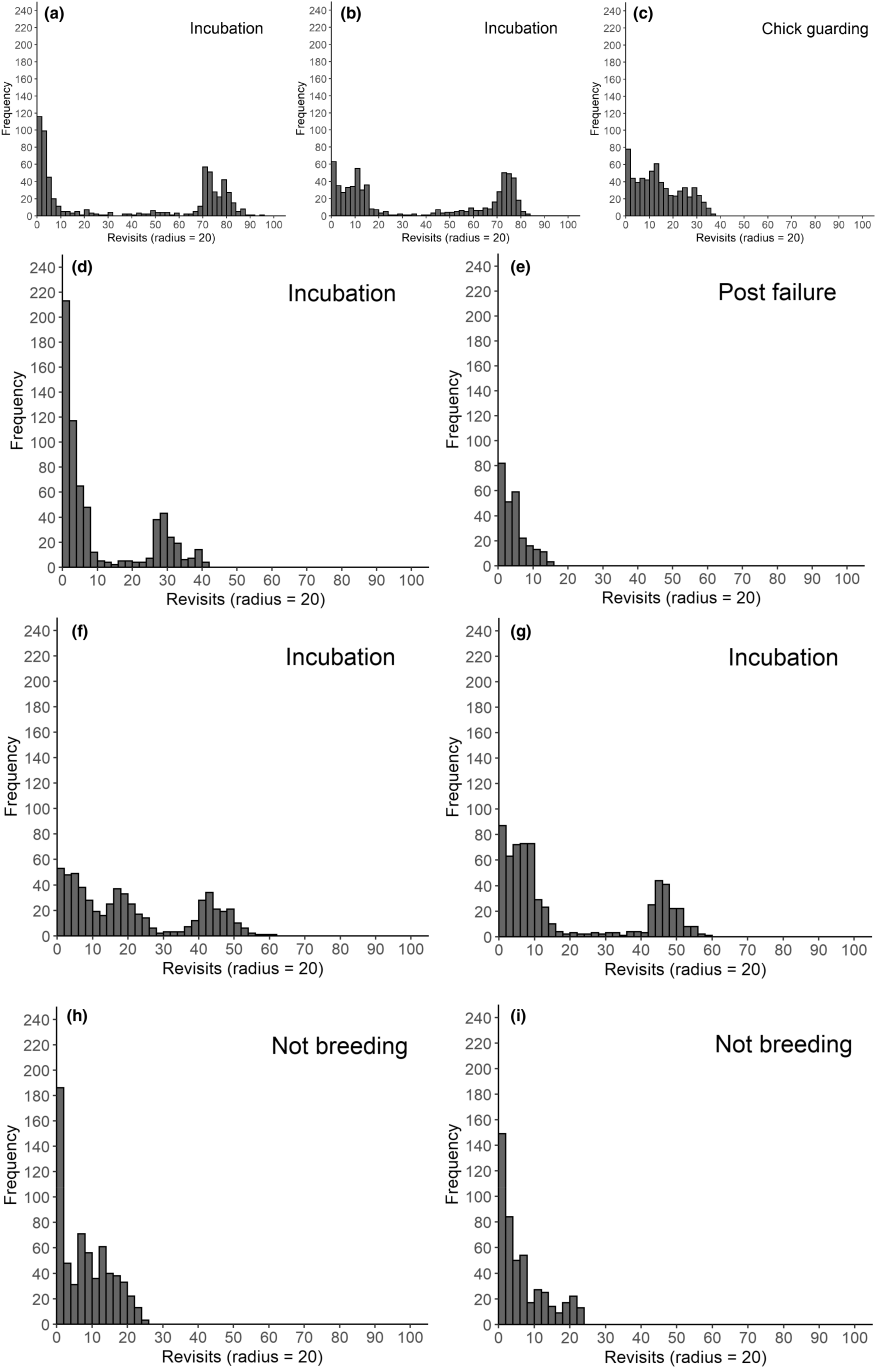
Weekly “recurse” frequency plots showing the frequency of revisits to sites within a moving 20 m radius for a male 002O_GO showing presumed (a) incubation from Week 21 to (b) Week 24 and (c) chick guarding in Week 25; a male 085O_RO whose presumed incubation failed between (d) Week 19 and (e) Week 20; a 2019 Anglesey female 007O_BW showing presumed incubation from (f) Week 18 to (g) Week 19; and a 2019 Snowdonia male 342W_BW that apparently did not make a breeding attempt for (h) Week 19 and (i) Week 20.

Based on the distribution patterns from the training data set above, we then identified initial periods of interest for the other tagged birds Curlew (example plots show in Figure 4) and then applying a 3‐day window (the smallest set of data analyzable) identified specific points of change between breeding status.

Table 2 summarizes the breeding attempts and outcomes predicted for each of the 23 birds following this assessment. Based on the analysis of this study, six of the birds showed no signs of breeding (26%), here no focal areas used or distribution patterns were identified to indicate incubation. We found patterns compatible with breeding attempts for the other 17 Curlew indicating they all made at least one breeding attempt (74% of all birds) with at least seven displaying movements compatible with two breeding attempts (30%) and one of those seven making a third attempt (4%) for a total of 25 attempts. At least 13 of the presumed first breeding attempts appeared to fail during incubation as did three of the seven's breeding attempts (16 of 25 attempts, 64%), although six birds lost their tags before incubation was apparently completed and thus these data were right censored. Of these birds, one lost its tag during its first presumed incubation, four during their second and one individual was on its third apparent incubation attempt.

**TABLE 2:** Breeding attempts and outcomes predicted using the R package “recurse” (Bracis et al., 2018) for Curlew tracked in North Wales between 2016 and 2019.

Tag ID	Sex	Year	Site	Nonbreeder	Breeding status at end of tagging	Nesting interval (I1,2,3)	Tagging data present for following statuses					
							PrB	I1	I2	I3	CG	PoB
285O_NO	M	2016	Snw	No	None	6	0	N	0	0	0	Y
301O_NG	M			No	CG	8	0	Y	0	0	OU	0
303O_NN	M			Yes	None		0	0	0	0	0	0
355O_RG	F	2018	Snw	No	None	4	Y	N	0	0	0	Y
341O_RR	M			Yes	None		0	0	0	0	0	0
352O_OO	M			No	I2	15, 7+	0	N	OU	0	0	0
339O_OW	M		Ang	No	None	30	0	Y	0	0	N	Y
340O_GG	M			Yes	None		0	0	0	0	0	0
016W_GO	F	2019	Snw	Yes	None		0	0	0	0	0	0
346W_OW	F			No	None	6	Y	N	0	0	0	Y
347W_OR	F			Yes	None		0	0	0	0	0	0
354W_BO	F			No	I2	5, 32+	Y	N	OU	0	0	0
342W_BW	M			Yes	None		0	0	0	0	0	0
348W_OG	M			No	None	8	Y	N	0	0	0	Y
351O_RW	M			No	None	6, 4+	Y	N	N	0	0	Y
709W_RB	M			No	None	12	Y	N	0		0	Y
007O_BW	M		Ang	No	I2	15, 10+	Y	N	OU	0	0	0
052O_GR	M			No	I3	5, 4, 4+	Y	N	N	OU	0	0
085O_RO	M			No	None	8	Y	N	0	0	0	Y
087O_RN	M			No	I2	13, 19+	Y	N	OU	0	0	0
211O_OW	M			No	I1	17+	Y	OU	0	0	0	0
002O_GO	M		Clw	No	CG	33	Y	Y	0	0	OU	0
165W_RR	M			No	No	6, 17	Y	N	N	0	0	Y

*Note*: The breeding status is abbreviated to CG, Chick guarding; I1, I2, I3, Incubating attempt; PrB, prebreeding; PoB, Post‐breeding. The areas are indicated by Ang, Anglesey; Clw, Clwydian Ranges; Snw, Snowdonia. Y = successfully completed this status (green), N = failure of this status (red), OU = outcome uncertain as tag fell off at this point (green).

Three birds, one in each year of the study, were thus presumed to have hatched chicks (18% of birds that attempted to breed and 13% of all birds). Two of these birds were either still chick guarding when their tags came off or they moved out of the study site. One bird lost its chicks and is the individual used for the training analysis. The other bird (002O_GO) was observed with chicks in the third week of June 2019, providing validation of the predicted status of this individual. Post‐breeding season sightings of large chicks suggested some of the birds whose tags fell off earlier in incubation may have also successfully raised fledglings.

One of the birds that successfully hatched chicks was tagged before it settled and started incubating and we observed 33 days of incubation behavior before we identified the chicks as hatching. Prebreeding behavior was observed in 13 of the 23 birds and post‐breeding behavior in nine. For birds with an observed start date of incubation that failed, we measured an average of 7.0 days (11 birds) before failure for incubation 1 and 7.3 days for incubation 2 (three birds).

The presumed (first and second) nest locations of two birds tagged together in 2019 on Anglesey (male 087O_RN and female 007O_BW) were identical, and it was thus assumed that these birds were a pair.

Based on the 25 predicted nesting (incubation) attempts and their fates, we estimated a daily nest survival rate over the incubation period of 0.935 (95% CI: 0.90, 0.96) and a point estimate of nesting success over the entire incubation period of 15.1%.

### 3.4 Data requirements for breeding stage identification

**FIGURE 5:**
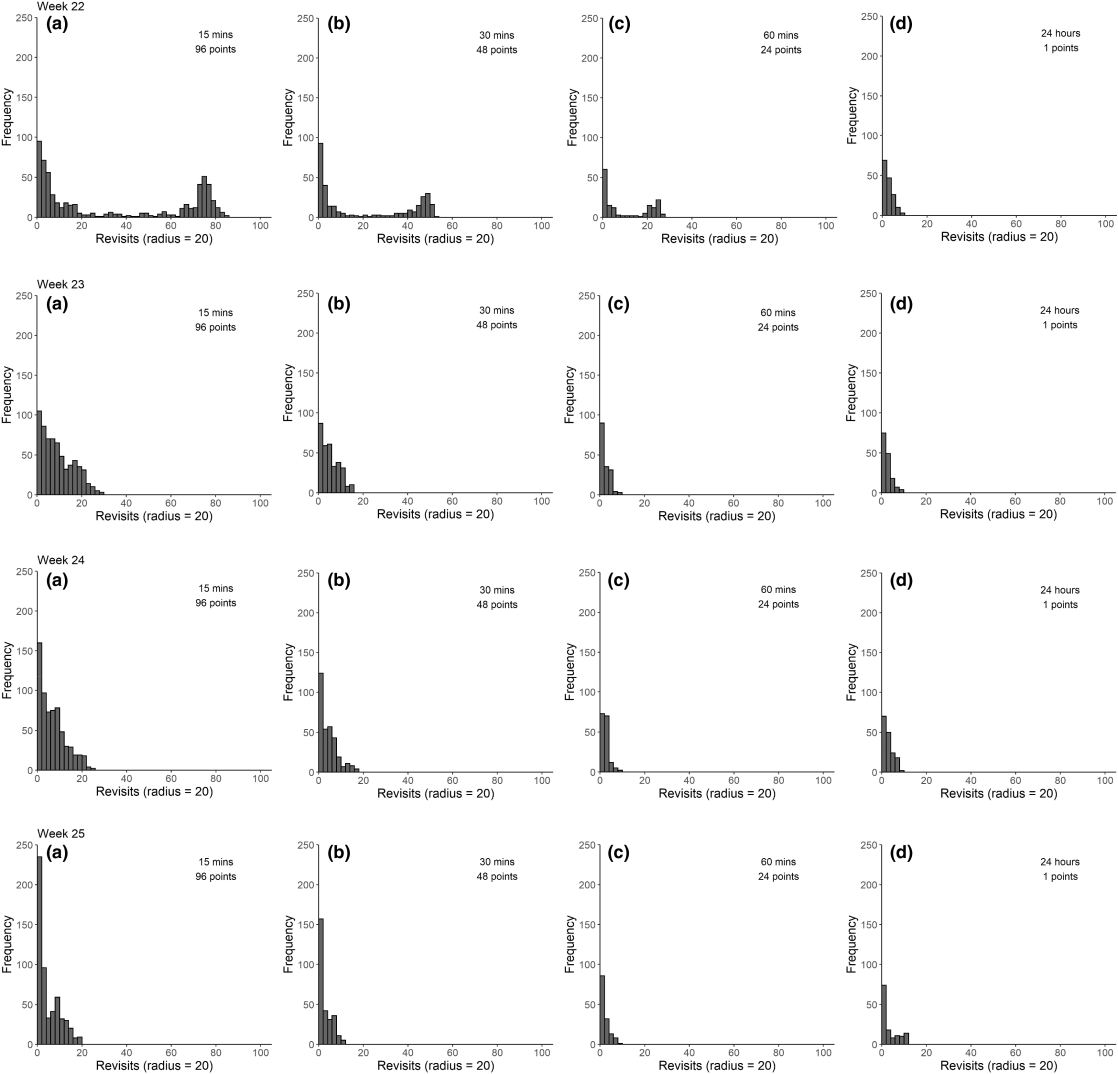
Weekly recurse frequency plots for a male Curlew (Numenius arquata) 339O_OW of known breeding status in 2018, showing the frequency of revisits to sites within a moving 20 m radius based on (a) the full data set collected at 15 min sampling intervals and reduced samples taken at (b) 30 min intervals, (c) 60 min intervals, and from (d) daylight mornings (08:00–13:59 UTC). Week 22 incubation; Week 23 chick guarding; Week 24 chick loss; Week 25 post‐breeding.

Frequency plots for male ID 339O_OW for 4 weeks of particular interest based on (a) the full data set collected at 15 min sampling intervals and reduced samples taken at (b) 30 min intervals, (c) 60 min intervals, and from (d) daylight mornings (08:00–13:59 UTC) are shown in Figure 5. The weeks of interest were week 22 when incubation was still occurring, week 23 when the switch to chick guarding occurred, week 24 when the chicks were lost and week 25 when the bird was no longer breeding.

The plots show that it was progressively less easy to detect reliable patterns in the data as sample sizes were reduced. Data sampled at 30‐min intervals showed similar patterns to the full data set collected at 15 min intervals. However, it was not possible to discern chick‐guarding behavior with data sampled at 60‐min intervals, while no detected patterns were apparent in data sampled from the morning.

**FIGURE 6:**
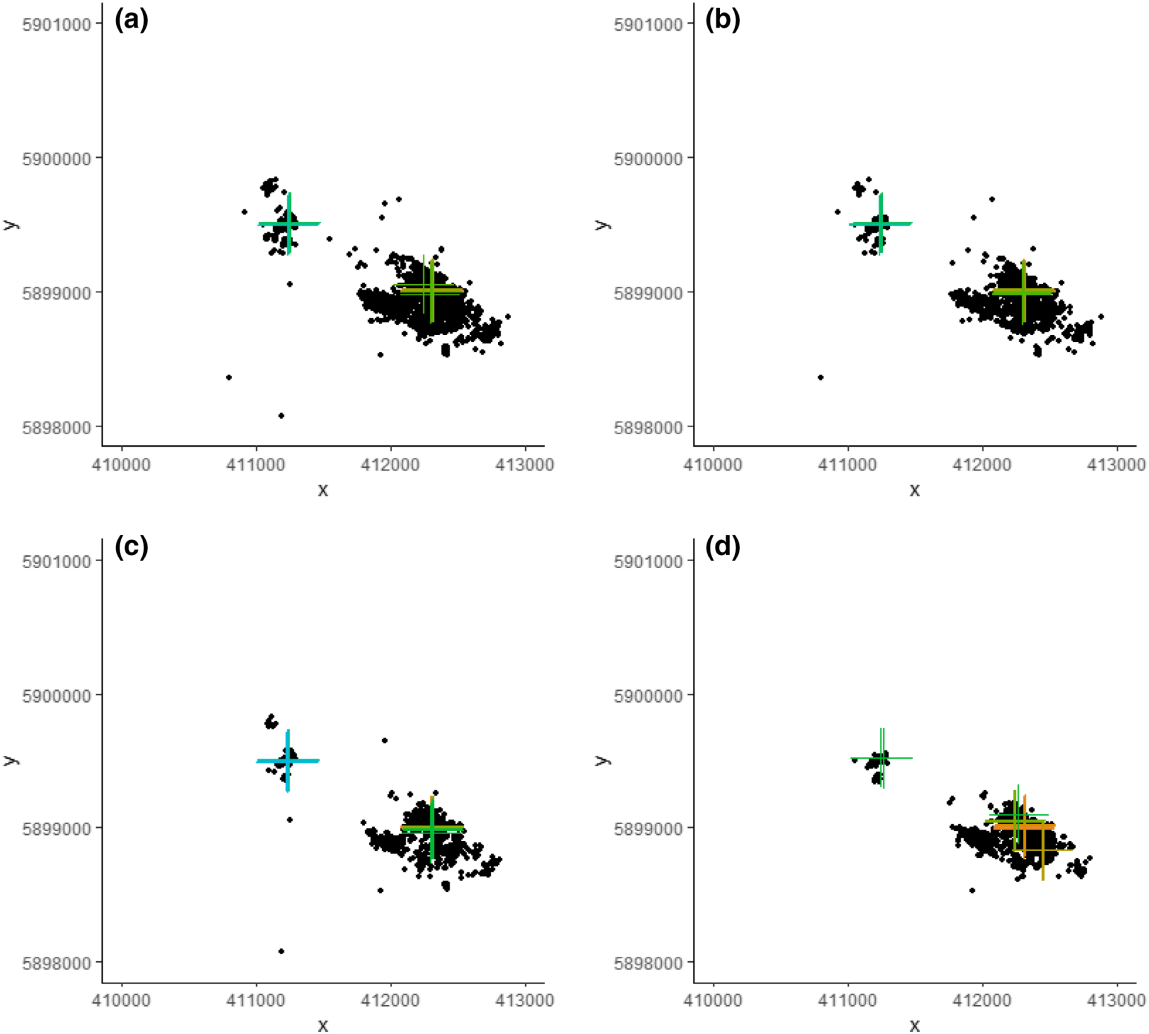
Recurse locational plots for a male Curlew (Numenius arquata) 339O_OW of known breeding status in 2018 based on (a) the full data set collected at 15 min sampling intervals and reduced samples taken at (b) 30 min intervals, (c) 60 min intervals, and from (d) daylight mornings (08:00–13:59 UTC). The colored crosses indicate the weekly focal locations (three identified per week for all bar the 2‐h period data sets where this is only one—requirements of package recommend three), and the black points indicate all the relocations in the data sets used. Green colors cover weeks 20–22, blue is for later weeks, and orange prior weeks.

With the full data set collected at 15 min sampling intervals, dates of change in breeding were visible with a minimum of 3 days of data. However, with data sampled at 30 and 60 min intervals, 6 and 9 days of data were needed, respectively. With data only sampled from the morning, 10 days of data were needed to detect patterns. While it was less easy to discern changes in breeding status when the data set was reduced in size and resolution, it was still possible to identify focal locations based on weekly data. Figure 6 shows the position of focal locations for male 339O_OW based on different sampling regimes.

## 4 DISCUSSION

Using the R package *recurse* (Bracis et al., 2018) and a training data set from an individual of known breeding status, we identified both the locations of nesting (incubation) attempts and the status of breeding of Curlews tagged with remote downloading GPS tags. While other studies have identified the nests from GPS tagging (Ewing et al., 2018), it is our understanding this is the first use of statistical preexisting recursion software to assess the frequency of revisits to particular locations to thus identify status through each breeding stage—prebreeding, incubation, chick guarding, and post‐breeding—for Curlew and thus breeding outcomes.

Curlew may be sensitive to disturbance and thus the study aimed to limit the number of monitoring visits, to limit the potential for nests to be deserted or for predators to be attracted to nests or chicks. Furthermore, the species may be cryptic in its behavior, meaning that detection of nests or adults with young can be problematic. Consequently, there was limited validation of the predictions provided by the analysis. Aside from the data used for training, an additional observation of a second bird with chicks provided validation of the predicted status of this individual. Nevertheless, we were able to detect patterns similar to those observed for the individual of known status across the data sets for other individuals providing some confidence of the reliability of this process.

Further research to provide validation of the approach would thus be of value. For example, nest cameras might be used to confirm dates of hatching or failure, and tagged adults relocated following hatching to determine if chicks are still alive. The apparent low nesting (incubation) success of Curlew in the present study, limited the information available during the chick‐guarding stage. Thus, use of the approach at other study sites would be particularly valuable in validating predictions during this breeding stage.

The approach used in this study, utilizing a preexisting statistical package (Bracis et al., 2018), provides a relatively quick method to identify breeding status in Curlew across multiple locations and years. It complements the practical side of fieldwork as changes in breeding status can be discerned from just 3 days of data in Curlew (~288 locations at 15 min intervals), meaning that additional observations or fast habitat sampling can be targeted if needed. It should be acknowledged that this process is unautomated in its current state and reliant on visual confirmation from an analyst requiring training and currently without directly quantifiable uncertainty, With increased applications to other tagging projects these limitations will be reduced.

The approach outlined here provides some similarities to other previous published studies that have considered how revisitation patterns might information on animal behavior (e.g., Picardi et al., 2020; Schreven et al., 2021). The *nestR* package (Picardi et al., 2022) developed by Picardi et al. (2020) to similarly detect nesting behavior in birds, also initially assesses revisitation patterns, but unlike the visual approach employed here, then uses an automated approach based on generalized metrics (but which can be tailored to different species) to provide predictions. As such, the *nestR* package may require a larger data set than the approach employed here, but provides a statistical classification of behavior associated with an assessment of uncertainty. Our method nevertheless differs from *nestR* in its ability to differentiate stages of breeding, in particular chick guarding behavior from incubation for Curlew which have precocial chicks unlike the altricial offspring of their study species. While *nestR* reduces the time needed to identify incubation bouts, without the specific knowledge of patterns of chick guarding, successful outcomes might potentially be mistaken for nest failure. The approach and patterns outlined here are specific to Curlew. However, for practitioners who have detailed knowledge of the behavior of their study species, and which may exhibit particular patterns of behavior—for example, as seen in the chick‐guarding stage for Curlew—that may not be correctly classified by more automated procedures, the visual approach presented here may thus provide a more practical solution for assessing the stage of breeding and consequently breeding outcomes.

The study also evaluated the temporal resolution of data required to discern breeding status in Curlew. With half hourly or hourly data, it was still possible to discern breeding status, but with more limited sampling, it became more difficult. There is a trade‐off between tag weight and sampling frequencies with GPS devices (Mitchell et al., 2019) and hence the minimum sampling frequencies suggested here may only be presently feasible for larger species, given recommendations that tags should represent no more than a given threshold (in the UK, 3%) of an individual's body weight (Geen et al., 2019). It was nevertheless still possible to discern focal locations with more reduced sampling.

Importantly, we can also use the results to identify breeding outcomes, length of incubation and time of day of hatching and failure. By identifying different status of breeding (i.e., prebreeding, incubation, chick guarding, post‐breeding), it is also possible to then assess how habitat use and requirements may change across the breeding cycle, enabling conservation management interventions to be better targeted. The composition of habitat in breeding areas is highly associated with nesting success in Curlew (Johnstone et al., 2017).

Our results suggest a low daily nest survival rate of 0.935 during incubation and that only a small proportion of individuals successfully raised young, concurring with other recent studies highlighting that population declines are likely to be driven by low levels of productivity. While the limitations of tag deployments to adult breeding birds meant that we could not confirm precisely how many chicks were fledged by tagged birds, best and worst‐case scenarios can be estimated (best case being birds still incubating/chick guarding at the end of tagging were successful). Standard monitoring guidelines for upland waders (Brown & Shepherd, 1993; Grant et al., 2000) recommend that potential breeding success is estimated based on numbers of territorial and alarm‐calling birds. Our work indicates that a proportion of birds did not make a breeding attempt even though they were territorial and responsive to play‐back during capture attempts. This would have important implications on nest productivity calculations and population viability analyses (Coulson et al., 2001).

There are many organizations working to reduce declines in Curlew populations across Europe both in the upland and lowland populations (Colwell et al., 2020; Douglas et al., 2021; Young et al., 2020). Direct and indirect conservation interventions do have some effects on Curlew populations (Franks et al., 2018) but are often time and labour intensive leading them to be spread few and far between (Wilson et al., 2020). Applying information gained from the analyses described in this paper that assesses productivity and locates breeding status specific habitats may reduce a high proportion of these issues.

Though the data set behind this study is relatively small, it has provided an approach that could be applied to future studies of Curlew both in Wales and elsewhere. Increases in sample size may allow full automation of breeding stage identification and reduce the time needed to manually identify when a different breeding status occurs. Previous tracking studies, for example, have shown how increased sampling of relocations (Girard et al., 2002) and larger numbers of tagged individuals (Lindberg & Walker, 2007) may improve the accuracy of home range estimation. By identifying a breeding status remotely, it is possible to undertake improved assessments of breeding success and more specific analysis of habitat use, enabling traditional fieldwork and management actions to be better targeted. We hope that the approach outlined will provide interest to other researchers studying similarly cryptic species and those that are sensitive to human presence and where traditional monitoring methods may pose a risk to the nesting success of the birds studied.

## AUTHOR CONTRIBUTIONS

**Katharine M Bowgen:** Conceptualization (equal); formal analysis (lead); methodology (lead); validation (lead); writing – original draft (lead). **Stephen Dodd:** Resources (equal); writing – original draft (supporting); writing – review and editing (equal). **Patrick Lindley:** Funding acquisition (lead); writing – review and editing (equal). **Niall H.K. Burton:** Funding acquisition (supporting); writing – original draft (supporting); writing – review and editing (equal). **Rachel C Taylor:** Conceptualization (equal); formal analysis (supporting); funding acquisition (supporting); methodology (supporting); resources (equal); supervision (lead); writing – original draft (supporting); writing – review and editing (equal).

## CONFLICT OF INTEREST

The authors declare that there are no conflicts of interest.

## Figures and Tables

**FIGURE A1:**
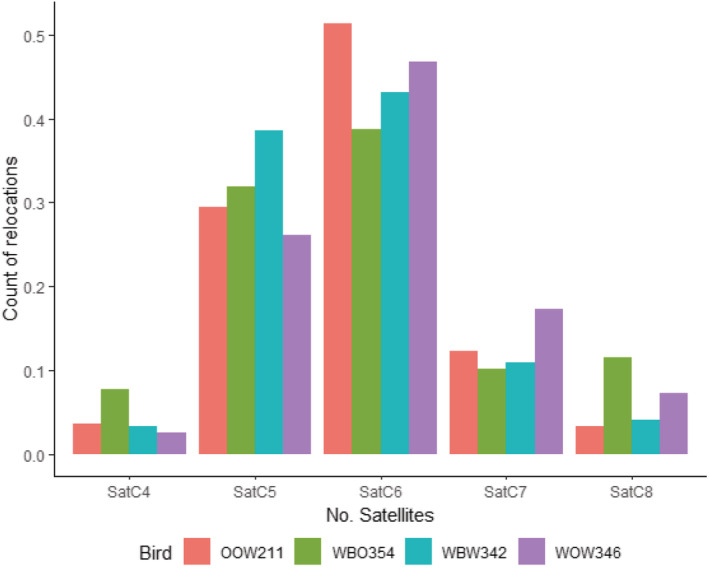
Count of relocations for each the recovered tags during their static periods categorized by number of satellites.

## Data Availability

The relocation data behind the analysis of this paper is jointly held by the British Trust for Ornithology, National Resources Wales, and the Royal Society for the Protection of Birds. All tracks used in this study have been uploaded to Zenodo (DOI: 10.5281/zenodo.7158225) under the title “Tracking data for breeding Curlew (Numenius arquata) in north Wales, UK.” Due to sensitivities around the locations of these breeding birds, these data sets have been spatially anonymised in terms of their location in Wales, UK. The joint funders are open to collaboration on the original data set and requests for access and collaboration can be made through Movebank (www.movebank.org) where the original track data sets are stored (Entitled: “Eurasian Curlew—breeding movements in north Wales”).

## APPENDIX 1

### Radius definition for recurse—Error margin assessment of recovered tags

To calculate revisits in the *recurse* package, a circle of a set radius is drawn around each point in a trajectory to allow the number of segments passing through this circle to be counted (Bracis et al., 2018). For the Curlew analysis presented in this paper, this radius was determined by the error margin of the relocations produced by the tags themselves.

In 2019, four tags were able to be recovered after being molted and contained several days of static data. These were from Anglesey (Tag 211O_OW) and Snowdonia (Tags 342W_BW, 346W_OW and 354W_BO), which fell off on the 18th May, 18th May, 14th May, and 8th June, respectively. The relocations were cropped to 1 day after they fell off (to avoid any prelim. movement) and the results for distance to the mean location (lat/long) of each tag per number of satellites used assessed. The distance was calculated using the *spDistsN1* function from the *sp* package (Pebsma & Bivand, 2005; Bivand et al., 2013).

The error margins in meters distance from the central location where the tag was recovered indicate that across all relocations and numbers of satellites (min four to max eight satellite connected) the mean distance for each tag ranged from 6.9 m to 54.1 m (Table A1). The highest errors were found for only four satellites connected at 37.8 m (24.8–54.1 m), while eight satellites reduced this to 7.3 m (6.9–8.1 m).

**TABLE A1:** Distance in meters from central location of recovered tag assessed by both number of satellites and individual tag identity.

Tag Number	4 Satellites	5 Satellites	6 Satellites	7 Satellites	8 Satellites
211O_OW	24.8	20.8	13.3	8.1	6.3
342W_BW	54.1	30.4	16.6	10.0	8.1
346W_OW	38.1	26.1	13.3	8.9	6.9
354W_BO	34.2	31.2	16.6	9.6	7.9
Averages	37.8	27.1	14.95	9.2	7.3
Average (full data set)	18.30				
Average (full data set with 5–8 satellites)	17.53				

Figure A1 shows the distribution of relocations by numbers of satellites attached indicating the low incidence of relocations made with <five and so assessing all satellites with >five satellites attached gave an average of 17.53 m though only slightly lower than the 18.30 m found including the 124 four satellite relocations. Using these results, an error margin of 20 m was deemed appropriate to use for this analysis.
